# A Case of Exomphalos

**Published:** 1864-02

**Authors:** H. Wythe Davis

**Affiliations:** Richmond, Va.


ART. II.—*A Case of Exomphalos.* By H. WYTHE DAVIS, M. D., Richmond, Va.

Teratology presents us with many cases of monstrosity, in which twin fœtuses are fused together to a greater or less extent, and more rarely we encounter cases of ectopia, in which from faulty development of the parietes of the body at the basial line, organs which should be internal are external, and which sometimes, from their bulk, complicate labor. Such examples are to be found in cases of exomphalos, where the liver and intestines have protruded through the umbilicus, Recently I have had the fortune to meet, in private practice, with a case in which nearly the whole of the abdominal parietes were absent, with protrusion of all the abdominal and the chief part of the thoracic viscera. Besides, there were other points of interest in the case, which, to a great extent, involved the life of the mother, not because of the bulk of the displaced abdominal and thoracic viscera of the child, but of the dislocation of the placenta, that organ being implanted partially over the os uteri.

I am desirous to communicate this case to the profession for two reasons; first, because it is important, and attended with danger; and secondly, because I can find no history of a similar deformity of fœtus in any obstetric author I have consulted.

November 22d, 1863—10 o'clock, A. M.—I was hastily sent for to visit Mrs. C—— in her first confinement. On my arrival I found the nurse, as well as the patient, much alarmed, there having occurred a very copious flooding. She was exhausted; pulse 120. I found that she had lost a considerable amount of blood, and the hemorrhage still very profuse. An examination per vaginum revealed the cause of the hemorrhage. The uterus was in a state of inertia, with the placenta partially implanted over the os uteri, which was about the size of a half dollar, but very dilatable. I ordered tincture ergot, which, with my hand in vagina, soon excited uterine action. I detached the placenta from its adhesion as far as I thought it safe for the preservation of the child, and was endeavoring to turn it aside for the descent of the fœtus, when suddenly and unexpectedly it was expelled. There was no longer any hemorrhage, as the fœtus now firmly engaged. I was unable to form a correct diagnosis of the presentation. By the touch I discovered organs (parts) resembling very much the feet, but the entire absence of the genitals and other important points of diagnosis prevented me from forming a positive opinion. The difficulty, too, was increased by a large mass on one surface, resembling, to the touch, the placenta, and on the opposite side a tumor, which felt something like the caput succedaneum. About one o'clock I delivered her of a seven months' fœtus, with the following deformities: The liver and heart were entirely exposed; the lungs partially protruded from under the ribs. The stomach and intestines, preserving their relative position, were covered with a delicate peritoneum Every portion of the alimentary canal could be distinctly defined. The termination of the rectum was exposed in front as other parts. On the lower part of the anterior surface was an imperfect cavity, which, from its situation and appearance, I would call the bladder; and communicating with this organ was another which, from its internal membrane, bore some resemblance to a uterus, to which, at its inferior part, was attached a body similar to a clitoris. These organs are very imperfectly developed, and could scarcely be recognized. On the posterior part was spina bifida occupying the lumbar region. The inferior limbs were reversed, the feet and knees looking backwards. Talipes varus of both feet, and anchylosis of the knee and ankle of the left limb. The funis, which measured only four inches, was inserted in the fœtus on the right side, about an inch from the vertebral column. The superior extremities and head were perfect in development.

Considering this a deformity of rare occurrence, I presented it to the Medical College of Virginia, where it may be seen.

